# TOR complex 2 localises to the cytokinetic actomyosin ring and controls the fidelity of cytokinesis

**DOI:** 10.1242/jcs.190124

**Published:** 2016-07-01

**Authors:** Karen Baker, Sara Kirkham, Lenka Halova, Jane Atkin, Mirita Franz-Wachtel, David Cobley, Karsten Krug, Boris Maček, Daniel P. Mulvihill, Janni Petersen

**Affiliations:** 1 School of Biosciences, University of Kent, Giles Lane, Canterbury, Kent CT2 7NJ, UK; 2 Faculty of Life Sciences, University of Manchester, Oxford Road, Manchester M13 9PT, UK; 3 Proteome Center Tübingen, Auf der Morgenstelle 15, Tübingen 72076, Germany; 4 Flinders Centre for Innovation in Cancer, School of Medicine, Flinders University, Adelaide, SA 5001, Australia; 5 South Australia Health and Medical Research Institute, North Terrace, PO Box 11060, Adelaide, SA 5000, Australia

**Keywords:** Rictor, TOR, TORC2, Myosin II, Myosin V, Acp1, CAPZA, *Schizosaccharomyces pombe*

## Abstract

The timing of cell division is controlled by the coupled regulation of growth and division. The target of rapamycin (TOR) signalling network synchronises these processes with the environmental setting. Here, we describe a novel interaction of the fission yeast TOR complex 2 (TORC2) with the cytokinetic actomyosin ring (CAR), and a novel role for TORC2 in regulating the timing and fidelity of cytokinesis. Disruption of TORC2 or its localisation results in defects in CAR morphology and constriction. We provide evidence that the myosin II protein Myp2 and the myosin V protein Myo51 play roles in recruiting TORC2 to the CAR. We show that Myp2 and TORC2 are co-dependent upon each other for their normal localisation to the cytokinetic machinery. We go on to show that TORC2-dependent phosphorylation of actin-capping protein 1 (Acp1, a known regulator of cytokinesis) controls CAR stability, modulates Acp1–Acp2 (the equivalent of the mammalian CAPZA–CAPZB) heterodimer formation and is essential for survival upon stress. Thus, TORC2 localisation to the CAR, and TORC2-dependent Acp1 phosphorylation contributes to timely control and the fidelity of cytokinesis and cell division.

## Abstract (precis)

**Highlighted Article:** A role for the TOR signalling pathway in regulating cytokinesis is identified. This myosin-dependent mechanism modulates the stability of the *S. pombe* CapZ complex to regulate actin function.

## INTRODUCTION

Target of rapamycin (TOR) signalling plays a key role in modulating the spatial and temporal control of cell growth in response to different environmental conditions. The TOR kinase forms two functionally distinct protein complexes, TOR complex 1 (TORC1) and TORC2 (Laplante and Sabatini, 2012). TORC1 and TORC2 are defined by unique components that are highly conserved across species. TORC1 contains regulatory associated protein of mTOR (RAPTOR, also known as RPTOR), and TORC2 contains Sin1 and rapamycin-insensitive companion of mTOR (RICTOR) (Wullschleger et al., 2006). It is rapamycin-sensitive TORC1 that is the major nutrient sensor, which integrates environmental cues with cell growth. TORC2 is regulated by different cues and exerts distinct functions (Laplante and Sabatini, 2012). Both TORC1 and TORC2 have been implicated in the control of cell migration and F-actin organisation (Liu and Parent, 2011). Inhibition of TORC1 with rapamycin prevents lamellipodia formation through a reduced expression of the small GTPase RhoA in mammalian cells (Liu et al., 2010). Similarly, TORC2 has been shown to play a key role in regulating the organisation and polarity of the actin cytoskeleton in *Saccharomyces cerevisiae*, *Dictyostelium discoideum* and mammalian cells (Jacinto et al., 2004; Lee et al., 2005; Schmidt et al., 1996).

The fission yeast, *Schizosaccharomyces pombe*, contains two TOR protein kinases, Tor1 and Tor2. The majority of TORC2 contains the non-essential catalytic kinase component Tor1 (Alvarez and Moreno, 2006; Hayashi et al., 2007; Matsuo et al., 2007). Importantly, all the functional specificities of TORC1 and TORC2 are conserved in yeasts. Cells lacking TORC2 components, such as Tor1, Sin1 or the fission yeast RICTOR homolog Ste20 (which is not a homolog of the budding yeast PAK-like kinase of the same name, and is hereafter denoted RICTOR^Ste20^) are larger than wild type, and are sensitive to heat, osmotic and oxidative stress. In addition these mutants are sterile as they are unable to undergo the G1 arrest that is an essential pre-requisite for mating in fission yeast (Kawai et al., 2001; Weisman and Choder, 2001). In fission yeast, F-actin cables are thicker than wild type and the cortical actin is atypically asymmetric in *tor1*-deficient mutants (Ikai et al., 2011; Matsuo et al., 2007). These distinctions prompted us to explore mechanisms by which TORC2 might impact upon actin cytoskeletal functions in fission yeast.

Here, we report a novel localisation of TORC2 to the cytokinetic actomyosin ring (CAR) where it has a role in moderating the successful completion of cytokinesis. We describe a mechanism by which TORC2 controls heterodimer formation between the actin-capping proteins Acp1 and Acp2 (the equivalent of mammalian CAPZA and CAPZB proteins, and are hereafter denoted CAPZA^Acp1^ and CAPZB^Acp2^, respectively) to regulate the actin cytoskeleton, and thereby the timely control of cytokinesis, to ensure survival upon changes to the extracellular environment.

## RESULTS

### TORC2 controls the integrity of the CAR

**Fig. 1.**
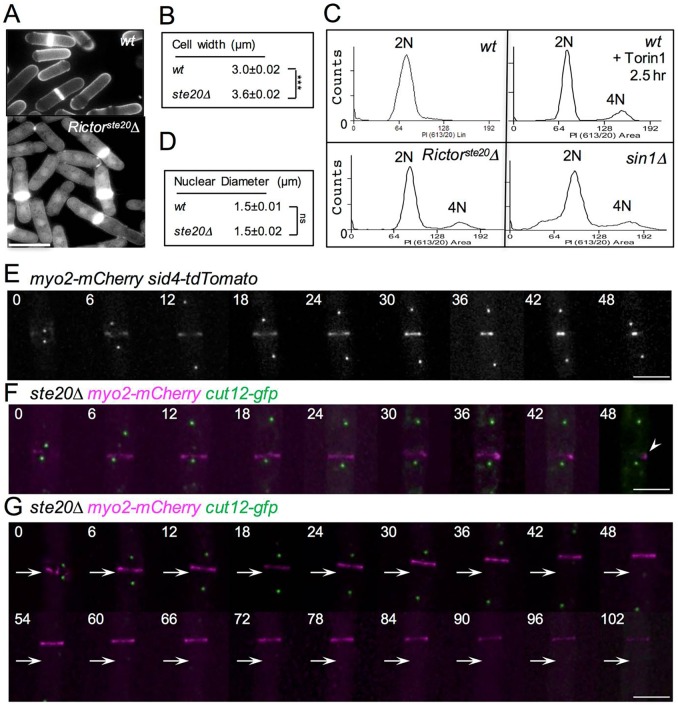
**TORC2-deficient mutants display defects in cytokinesis and cell fission.** (A) Early exponential wild-type (*wt*) and *Rictor^ste20^*Δ (*ste20*Δ) prototroph cells were stained with Calcofluor to visualise the division septa. Scale bar: 10 µm. These cells, as well as *sin1*Δ cells and cells treated with Torin1, were (C) processed for flow cytometry analysis to measure DNA content. Mean cell width (B) and nuclear diameter (D) were each determined from counting 300 wild-type and *Rictor^ste20^*Δ cells in mid-log culture. ***P<0.001, ns, not significant (Student's *t*-test). (E) A montage of time-lapse images showing red fluorescence from *myo2–mCherry sid4–tdTomato* cells undergoing cell division. Cells illustrating timing of CAR formation and constriction in relation to SPB segregation in wild-type cells are shown. (F,G) Equivalent montages of time-lapse images of mCherry (magenta) and GFP (green) fluorescene from *myo2–mCherry cut12–gfp* *Rictor^ste20^*Δ cells. In a large proportion of anaphase *Rictor^ste20^*Δ cells the CAR was seen to either collapse (arrowhead, F) or drift along the cortex towards one end of the cell (arrows, G). Scale bars: 5 µm.

In order to examine the potential roles that TORC2 might have during actin-dependent cell growth and division (Lancaster and Baum, 2014), we undertook a phenotypic analysis of fission yeast cells lacking RICTOR^Ste20^ (denoted *Rictor^ste20^*Δ), the core TORC2-specific component. The main catalytic component of TORC2 is the Tor1 protein kinase (Alvarez and Moreno, 2006; Hayashi et al., 2007; Matsuo et al., 2007); however, cells lacking Tor1 are capable of incorporating Tor2 into the TORC2 complex (Matsuo et al., 2007; Hartmuth and Petersen, 2009). In contrast, deleting RICTOR^Ste20^ from the genome specifically ablates TORC2 function within the cell. *Rictor^ste20^*Δ cells are elongated and display an altered cell morphology, with a cell diameter significantly greater than otherwise isogenic wild-type controls (Matsuo et al., 2007; Hartmuth and Petersen, 2009; Fig. 1A,B; Fig. S1A). Increased cell diameter and cell length is normally associated with cells in the diploid lifecycle. Fluorescence-activated cell sorting (FACS) analysis of TORC2-deficient mutants or wild-type cells after an acute treatment with Tor inhibitor 1 (Torin1) (Atkin et al., 2014) revealed the major 2N peak that is normally associated with wild-type haploid fission yeast cells (Fig. 1C), along with an additional minor 4N peak (Fig. 1C). Cells in the diploid lifecycle have significantly larger nuclei than haploid cells (Neumann and Nurse, 2007). The nuclei diameter were equivalent in wild-type and *Rictor^ste20^*Δ cells (Fig. 1D) indicating that the TORC2-deficient mutants are haploid cells with altered cell size. The minor 4N peak observed by FACS might be brought about by a small population of diploid cells. Alternatively, this small peak could represent cells in the haploid life cycle delayed in cell division but which have completed the next S-phase. Division septa were often aberrant and misplaced, with the septum located away from the cell equator in 55% of *Rictor^ste20^*Δ cells [these included septa positioned more than 5% away from the cell equator (of the distance spanning the cell end to the cell equator)]. In contrast, none of the septa were misplaced in all observed wild-type cells. Finally, elevated levels of new cell wall material was observed at the cell equator in the absence of TORC2 function (Fig. 1A; Fig. S1A). Taken together, these data indicate that TORC2-deficient haploid cells display defects in cytokinesis and cell fission.

Visualisation of septal material in *Rictor^ste20^*Δ cells revealed that a large proportion of cells had misplaced or aberrant septa (Fig. 1A; Fig. S1A). *myo2–mCherry* cells were used to follow CAR localisation and dynamics. *myo2–mCherry* cells containing functional TORC2 displayed growth characteristics and genetic interactions equivalent to the wild-type *myo2^+^* allele (Fig. 1E; Movie 1). In contrast, time-lapse imaging of *myo2–mCherry Rictor^ste20^*Δ cells revealed that TORC2-deficient cells remained in the phase of the cell cycle dedicated to cytokinesis for a prolonged period. As well as displaying delayed constriction, the CAR was seen to either split or collapse (39% of cells) (Fig. 1F, Movie 2) or slide along the cortex (11% of cells) (Fig. 1G). This is consistent with our observation that a substantial proportion of septa in *Rictor^ste20^*Δ cells formed septum that were more than 5% of the cell length away from the centre of the cell (Fig. S1A). In a small proportion (∼ 2%) of these *Rictor^ste20^*Δ cells, the spindle was seen to extend from one side of the misplaced CAR (Fig. S1B), these cells are likely to contribute to the 4N peak observed by FACS (Fig. 1C). In addition, *Rictor^ste20^*Δ cells often failed to integrate medial Myo2 foci into a stable CAR over an extended period of several hours without inhibiting cell growth (<5% of cells) (Movie 3), and were also seen to complete CAR constriction, and then reform at the division site before dividing in two (7% of cells) (Movie 4). Consistent with this observation, anti-tropomyosin antibody immunofluorescence staining of actin filaments in *Rictor^ste20^*Δ cells revealed defects in actin ring function during cytokinesis, with divided cells possessing an unconstructed CAR structure at the cell end at which cell division had just occurred (Fig. S1C). Thus, TORC2 function is not only required to maintain normal cell size, but also plays a crucial role in regulating the timing of CAR formation and maintaining its subsequent integrity during cell division.

### TORC2 interacts with and localises to the CAR during ring constriction

**Fig. 2.**
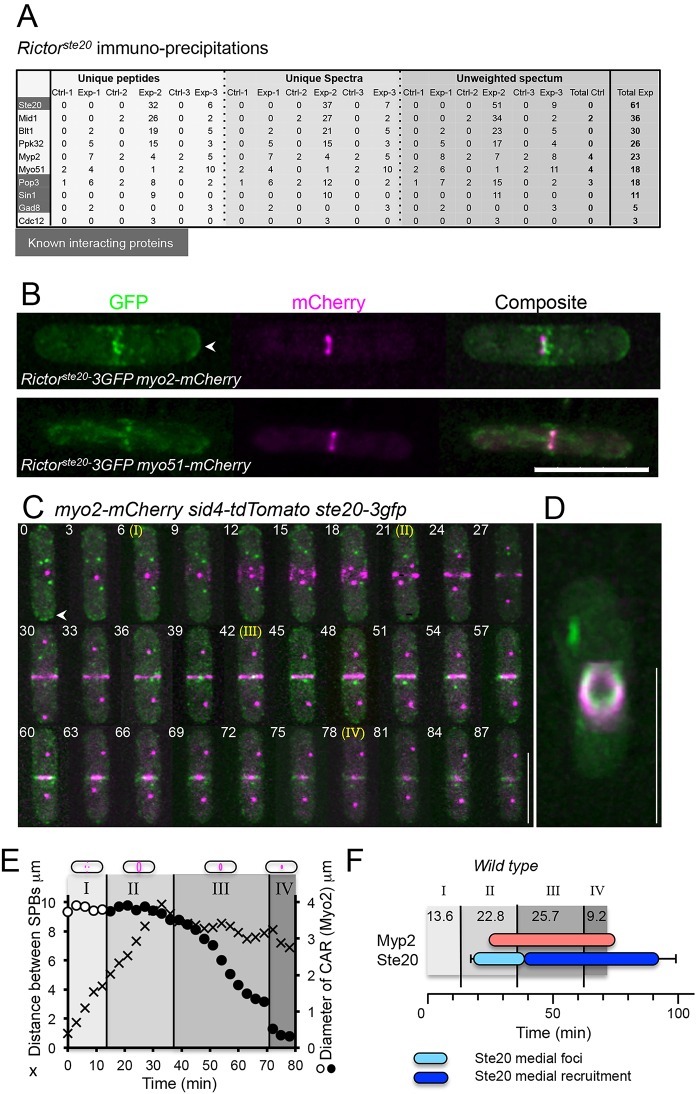
**TORC2 interacts with and localises to the CAR during ring constriction.** (A) Summary of mass spectrometry analysis of three independent experiments of control (Ctrl, no anti-Tor1 antibodies added) and Tor1 immunoprecipitations (Exp), each purified from 20-l cell cultures. (B) Micrographs of RICTOR^Ste20^ (green) and Myo2 or Myo51 (magenta) signal from mitotic *RICTOR^Ste20^*–*3GFP* *myo2–mCherry* cells or *RICTOR^Ste20^*–*3GFP* *myo51–mCherry* cells. (C,D) Maximum projections of 21-slice *z*-stacks from a timecourse of mitotic *myo2–mCherry sid4–tdTomato* *RICTOR^Ste20^*–*3GFP* cells reveals that RICTOR^Ste20^ foci (green) are recruited to the cell equator after SPB (magenta) separation and Myo2 ring (magenta) formation has occurred (frame 1–4 with non-separated SPBs represent interphase cells) (C) and coalesce to form a ring during CAR constriction. (D) Micrograph of mCherry and GFP signal from a *RICTOR^Ste20^*–*3GFP* *myo2–mCherry* cell illustrating TORC2 association with the CAR. In B and C, arrowheads highlight RICTOR^Ste20^ localisation at cell tips. (E) The timing of key events during CAR formation and constriction [(I) Myo2 foci (empty circles) recruit to the cell equator; (II) Myo2 foci coalesce to form a CAR (filled circles); (III) the CAR constricts until, (IV) it reaches a diameter of 0.5 µm or less] were determined in relation to nuclear division (crosses, distance between SPBs) in wild-type. (F) The timing of the appearance of Myp2 ring (red bar) and RICTOR^Ste20^ medial foci (light blue bar) or RICTOR^Ste20^ ring recruitment (dark blue) were determined in wild-type strains in relation to the events determined in E. Scale bars: 10 µm.

In order to explore how TORC2 affects the integrity of the CAR, we undertook a proteomic-based analysis to identify proteins that co-purified with the main TORC2 catalytic component, Tor1. Mass spectrometry of Tor1 immuno-precipitates from 20 l of early-log phase culture identified three known core TORC2 components (RICTOR^Ste20^, Sin1 and Pop3) as well as the TORC2 substrate Gad8 (Fig. 2A). Intriguingly, as well as co-purifying with Cdc12, a formin required for nucleation of tropomyosin-stabilised actin filaments within the CAR, in three independent experiments Tor1 co-purified with the class II and V myosin heavy chains, Myp2 and Myo51, both of which are core components of the actomyosin cytoskeleton and cytokinetic division machinery (Fig. 2A).

These links between TORC2 and control of cytokinesis prompted us to re-visit the localisation studies reporting GFP-tagged RICTOR^Ste20^ (RICTOR^Ste20^–3GFP) recruitment to the plasma membrane (Tatebe et al., 2010). Live-cell imaging of *myo2–mCherry RICTOR^Ste20^–3GFP* and *myo51–mCherry RICTOR^Ste20^–3GFP* cells revealed that TORC2 colocalised with each myosin heavy chain at the CAR (Fig. 2B). To correlate the timing of TORC2 reorganisation and recruitment to the contractile apparatus with spindle dynamics and CAR formation, RICTOR^Ste20^–3GFP dynamics was examined in cells expressing an mCherry-labelled allele of the essential class II myosin *myo2* and tdTomato-labelled version of the essential spindle pole body (SPB) component Sid4 (*myo2–mCherry sid4–tdTomato* cells) (Fig. 2C, Movie 5). Upon entry into mitosis, Myo2 was recruited to foci at the cell equator, and the two separate SPBs were juxtaposed the elongating mitotic spindle until metaphase (Fig. 2C–E, phase I; Fig. S1D). At the onset of anaphase, the spindle elongates and Myo2 foci coalesce to form the CAR (phase II) (Mulvihill and Hyams, 2002; Wu et al., 2003). It is at this time that foci of RICTOR^Ste20^–3GFP localised to the CAR, where they remained through its subsequent constriction (phase III), and disassembly (phase IV), when they were recruited to the ensuing new cell end (Fig. 2C,F; Movie 5). Thus, TORC2 localises to the CAR during mitosis where it interacts with Cdc12, Myp2 and Myo51, key regulatory components of CAR formation and function.

### Myosin V and myosin II regulate RICTOR^Ste20^ recruitment at the CAR

**Fig. 3.**
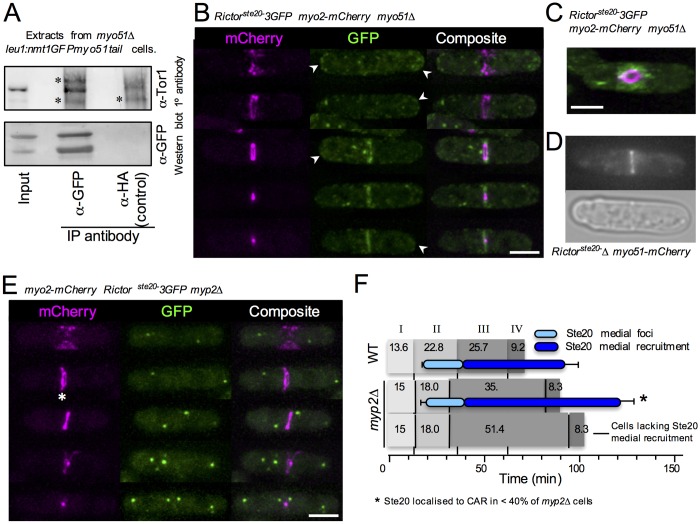
**Myosin II and V interact with and regulate RICTOR^Ste20^ localisation at the CAR.** (A) Extract and subsequent anti-GFP and anti-HA (control) immunoprecipitates from *S. pombe* cells expressing a GFP-tagged Myo51 cargo-binding-tail domain fusion protein were subject to anti-Tor1 (upper panels) and anti-GFP (lower panels) antibody western blot analysis. The asterisks denote background bands. (B–E) Micrographs of mCherry and GFP signals in cells with the indicated genotype. The asterisk highlights rings split in two. Arrowheads highlight RICTOR^Ste20^ localisation at cell tips. (F) The timing of the appearance of RICTOR^Ste20^ medial foci (light blue bars) or RICTOR^Ste20^ ring recruitment (dark blue) were determined in wild-type and *myp2*Δ strains in relation to the events determined in Fig. 2E. Scale bars: 5 µm.

We next decided to investigate the physical interaction between RICTOR^Ste20^ and the myosins, Myp2 and Myo51, to explore whether these actin-associated motor proteins play a role in recruiting TORC2 to the cell equator during cytokinesis. The class V myosin, Myo51, plays key roles in regulating CAR function and dynamics (Bezanilla et al., 1997; Win et al., 2001). Fission yeast contains two myosin V proteins, Myo51 and Myo52. The minor myosin V isoform, Myo51, localizes to the CAR (Motegi et al., 2001; Win et al., 2001) and is required for correct CAR formation (Fig. S1E). Small-scale immunoprecipitation confirmed the physical association between TORC2 and the cargo-binding domain of Myo51, as Tor1 co-purified with the Myo51 tail fused to GFP (Doyle et al., 2009) (Fig. 3A). This confirmation, combined with our observation that Myo51–mCherry colocalised with RICTOR^Ste20^−3GFP during cytokinesis (Fig. 2B) provides strong evidence that Myo51 interacts with TORC2 during cytokinesis. Consistent with this finding, RICTOR^Ste20^−3GFP failed to localize to the ring in the absence of Myo51 (Fig. 3B,C), and localised instead to the septum as it forms around the outside edge of the constricting CAR (Fig. 3C). Removal of the second myosin V homologue through deletion of the *myo52*^+^ gene had no discernible impact upon RICTOR^Ste20^−3GFP distribution (data not shown). In contrast, RICTOR^Ste20^ function was not required for Myo51 recruitment to the CAR (Fig. 3D). Thus, the recruitment of TORC2 to the CAR is dependent upon the myosin V motor Myo51, with which it physically associates.

Like Myo51, the class II myosin Myp2 physically interacts with Tor1 (Fig. 2A) and plays a role in maintaining the integrity of the CAR during cytokinesis. Cells lacking Myp2 display cytokinesis defects (Bezanilla et al., 1997; Mulvihill et al., 2000) similar to those observed here for *Rictor^ste20^*Δ cells. For example, the CAR sometimes split in two, and each half constricts independently of each other (Fig. 3E, asterisk) (Mulvihill and Hyams, 2003). Interestingly, in the majority (>60%) of cells lacking Myp2, RICTOR^Ste20^ did not localise to the cell equator (Fig. 3E,F). Intriguingly, Myp2 was also required for RICTOR^Ste20^−3GFP localisation to cell poles (Fig. 3E, compare lack of cell pole localisation with that observed in *myp2^+^* cells, highlighted by arrowheads in Figs 2 and 3). Why this CAR component should affect the cortical TORC2 localisation is currently unclear; however, western blot analysis confirmed persistence of RICTOR^Ste20^ protein in the *myp2*Δ cells (data not shown). To further investigate and confirm the dependence of TORC2 upon Myp2 for localisation and interaction with the CAR, we used time-lapse imaging to characterise the relative timing of spindle dynamics, CAR formation and TORC2 localisation to the cell equator. Time-lapse imaging of more than 30 prototroph wild-type cells revealed CAR constriction (phase III) lasted 25 min (Fig. 3F). In contrast, this event took twice as long in cells lacking Myp2 (50 min), confirming the role of this protein in modulating CAR constriction (Bezanilla et al., 1997; Mulvihill et al., 2000; Huang et al., 2012). RICTOR^Ste20^−3GFP failed to localise to the cell equator in the majority (∼60%) of *myp2*Δ cells, and these cells took almost twice as long as equivalent *myp2^+^* cells to complete CAR constriction (phase III) (Fig. 3F; Fig. S1D). However, RICTOR^Ste20^ localised correctly in the remaining ∼40% of *myp2*Δ cells, and phase III took ∼50% longer to complete than wild type (Fig. 3F; Fig. S1D). Taken together, our observations are consistent with both class II and V myosins co-purifying with TORC2, and playing a role in regulating TORC2 localisation to the CAR.

### TORC2 regulate Myp2 CAR localisation and the timing of CAR constriction

**Fig. 4.**
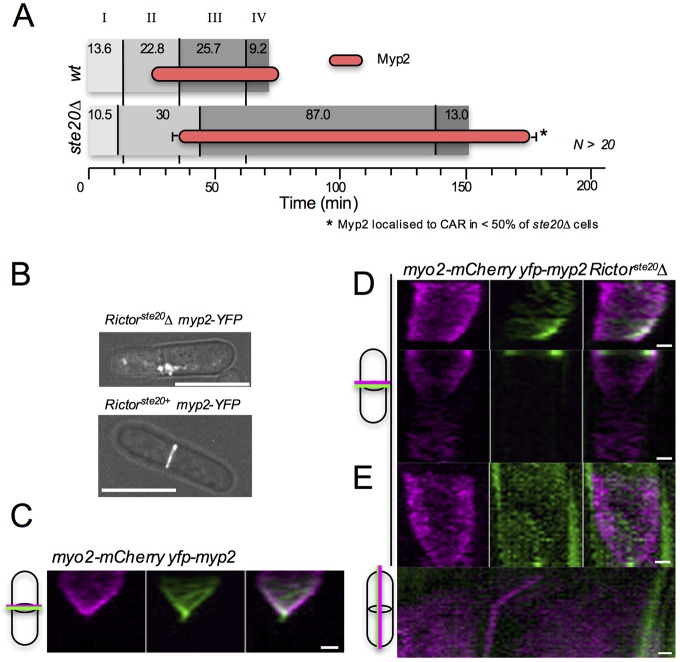
**Myp2 and TORC2 localisation to the actomyosin ring is co-dependent.** (A) The timing of Myp2 ring recruitment (red bars) was determined in wild-type and *Rictor^ste20^*Δ strains in relation to the events determined in Fig. 2E. (B) Composite micrographs of YFP and phase signal in *yfp–myp2 Rictor^ste20+^* and *yfp–myp2 Rictor^ste20^Δ* cells. (C–E) Kymographs generated from 30 maximum projections of time-lapse images (3 min/frame) of (C) *myo2–mCherry YFP–myp2* and (D) *myo2–mCherry YFP–myp2 Rictor^ste20^*Δ cells illustrating that TORC2 is required for Myp2 to remain at the CAR. (E) Time-lapse kymographs of the perpendicular (upper panels) and longitudinal (lower panels) axes of a *yfp–myp2 Rictor^ste20^*Δ cell in which Myp2 signal is lost from the cell equator and the Myo2-containing CAR slides along the cell cortex before constricting. In C–E, cartoons illustrate orientation and origin of kymograph axes. Scale bars: 5 µm (B); 1 µm (C–E).

We further explored the link between TORC2 and the timing of cytokinesis by visualising cytokinetic ring dynamics using the myosin II motor proteins, Myo2 and Myp2, in *Rictor^ste20^*Δ cells. Time-lapse imaging of more than 30 *Rictor^ste20^*Δ cells revealed that, of those cells which formed a CAR, constriction and disassembly (Fig. 4A, phase III and IV) took on average more than three times longer than in wild-type cells (Fig. 4A; Fig. S1A). RICTOR^Ste20^ only localised to the CAR in 40% of *myp2*Δ cells (Fig. 4), likewise Myp2 recruitment to the CAR was frequently aberrant in *Rictor^ste20^*Δ cells (Fig. 4B,D) indicating a co-dependency. Although Myp2 was recruited to the CAR 10.3 min after its formation (phase II) in wild-type cells, and remained there throughout CAR constriction (Fig. 4A,C; Movie 6), Myp2 CAR recruitment was extremely transient in the majority of mitotic *Rictor^ste20^*Δ cells lacking TORC2 function (Fig. 4D). Crucially, in *Rictor^ste20^*Δ cells in which Myp2 localised to the CAR, the ring remained un-constricted for a long period (Fig. 4D,E), and the contractile apparatus was often observed sliding along the length of the cell (Fig. 4D,E). Therefore, TORC2 not only co-purifies with regulators of cytokinesis, but ablating TORC2 function leads to defects in CAR formation and severe delays in its constriction (Fig. 4A; Fig. S1A).

### TOR-dependent phosphorylation on serine residues 172 and 189 of Acp1

To determine whether the TORC2-interacting CAR components (Fig. 2A) are substrates of the complex we examined data from a stable isotope labelling by amino acids in cell culture (SILAC) mass spectrometric screen for phospho-peptides that showed differential phosphorylation upon Torin1 inhibition of TOR function (Atkin et al., 2014). With the exception of Ppk32 and Myo51, phosphorylation sites were identified in all co-purifying proteins. Crucially, none of these sites were regulated by TOR signalling, as they remained unchanged following TOR inhibition by Torin1 (Fig. S1F).

**Fig. 5.**
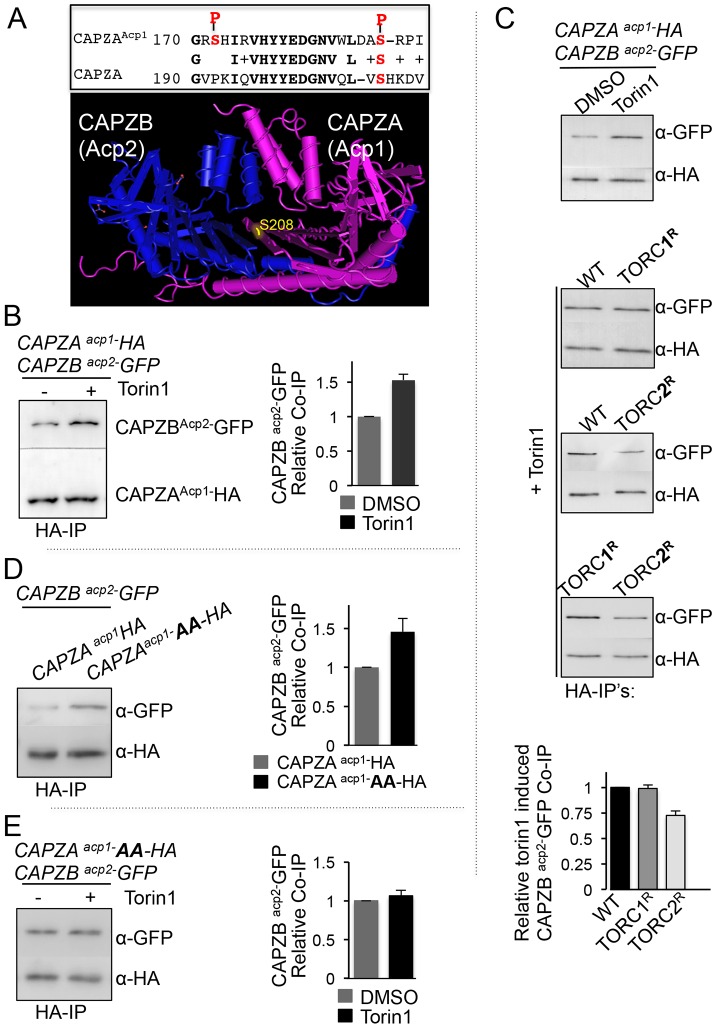
**TORC2 regulates CAPZA ^Acp1^–CAPZB^Acp2^ heterodimer formation.** (A) Alignment of the phosphorylated region of *S. pombe* Acp1 and the human CAPZA homologues. Conserved residues are highlighted in bold and the two TOR-dependent phosphorylation sites are shown in red. The position of the conserved phosphorylated serine 208 (in yellow) is shown on the crystal structure of CAPZA and CAPZB (Yamashita et al., 2003). (B–D) Anti-GFP (upper panels) and anti-HA (lower panels) antibody western blots of CAPZA^Acp1^−HA immunoprecipitations (HA-IPs) from indicated strains in the absence or presence of the TOR inhibitor Torin1 (25 µM) (TORC1^R^=*tor2.G2037D* TORC2^R^=*tor1.G2040D*). (B–E) To ensure the measured relative levels of Acp1 and Acp2 within anti-HA immunoprecipitates were quantifiable, western blot membranes of immuno-precipitates were cut in half and the separate portions were subsequently probed with anti-GFP (upper panels) and anti-HA (lower panels) antibodies.

Importantly, cytokinesis and the dynamics of actin polymers are regulated, in part, by a heterodimeric complex consisting of the actin-capping proteins Acp1 and Acp2 (homologues of human CAPZA and CAPZB proteins; hereafter denoted CAPZA^Acp1^ and CAPZB^Acp2^, respectively), which binds to and stabilises the barbed end of actin structures (Yamashita et al., 2003). Interestingly, the TOR-phosphorylation-dependent SILAC screen revealed that CAPZA^Acp1^ displayed differentiation phosphorylation on serine residues 172 and 189 (Fig. 5A; Fig. S2A,B) in Torin1-treated cells. However, the sequence that serine 172 and 189 lies within does not conform to the TOR consensus phosphorylation site (Hsu et al., 2011), suggesting that phosphorylation of CAPZA^Acp1^ is dependent on TOR rather than being a direct target. The Psk1 and Gad8 kinases are two known TORC1 and TORC2 substrates and effector kinases (Pearson and Kemp, 1991). Both Gad8 and Psk1 are members of the AGC family of kinases. However, the CAPZA^Acp1^ phosphorylation sites do not conform to the AGC kinase consensus site either. It is therefore possible that the TOR-dependent CAPZA^Acp1^ phosphorylation is regulated through an as yet unidentified signalling pathway. Interestingly, serine 189 is conserved in the mammalian CAPZA homologues (serine 208) (Fig. 5A), suggesting that TOR-dependent phosphorylation of CAPZA could be conserved in metazoan systems.

### TORC2-dependent phosphorylation alters CAPZA^Acp1^–CAPZB^Acp2^ heterodimer formation

The crystal structure of the human CAPZA–CAPZB heterodimer (Yamashita et al., 2003) reveals the CAPZA^Acp1^ serine 189 equivalent within human CAPZA maps to the CAPZA–CAPZB interface (Fig. 5A), and therefore its phosphorylation is likely to have an impact upon heterodimer formation and function. In order to assess the impact TOR-dependent CAPZA^A^^cp1^ phosphorylation had upon CAPZA^Acp1^–CAPZB^Acp2^ heterodimer formation we undertook equivalent co-immunoprecipitation assays in wild-type cells treated with either Torin1, to inhibit TOR signalling (Atkin et al., 2014), or DMSO. Although CAPZA^Acp1^–HA was seen to co-purify with CAPZB^Acp2^−GFP in wild-type cells, this interaction was substantially enhanced by treatment with Torin1 (Fig. 5B). A conserved mutation within the ATP-binding pocket of genes encoding for either of the two fission yeast Tor kinases (*tor2.G2040D tor1.G2037D*; denoted *TORC1^R^* and *TORC2^R^*, respectively)* * specifically confers TORC1- or TORC2-dependent resistance to Torin1-induced inhibition (Atkin et al., 2014). These mutants can be used to establish whether the Torin1-induced effects are brought about by off-target effects. Torin1 enhanced CAPZA^Acp1^–CAPZB^Acp2^ heterodimer formation in wild-type and the *TORC1^R^* mutant (Fig. 5C). In contrast, heterodimer formation was not enhanced in the *TORC2^R^* mutant upon Torin1 treatment. Therefore, as TORC2 activity in the *TORC2^R^* mutant is resistant to the effects of Torin1, this result indicates that it is the specific inhibition of TORC2 that enhances the affinity between CAPZA^Acp1^ and CAPZB^Acp2^.

To establish whether TORC2-dependent CAPZA^Acp1^ phosphorylation alters the stability of the CAPZA^Acp1^–CAPZB^Acp2^ heterodimer we generated strains in which the endogenous *CAPZA^a^^cp1^*^+^ locus was mutated to encode for CAPZA^Acp1^ protein in which serine 172 and 189 had been replaced with alanine to mimic an unphosphorylated serine (denoted CAPZA^Acp1^-AA). CAPZA^Acp1^–CAPZB^Acp2^ co-immunoprecipitations were repeated using extracts from *CAPZA^acp1^-AA* cells and the CAPZA^Acp1^-AA mutant protein was seen to have an increased affinity for CAPZB^Acp2^ compared to wild type (Fig. 5D). Crucially, the Torin1-induced stabilisation of the CAPZA^Acp1^–CAPZB^Acp2^ heterodimer was abolished with the *CAPZA^acp1^-AA* allele (Fig. 5E). Thus, these data indicate that TORC2-dependent phosphorylation of CAPZA^Acp1^ serine 172 and 189 regulates CAPZA^Acp1^–CAPZB^Acp2^ heterodimer formation.

### The CAPZA*^acp1^*-AA–CAPZB*^Acp2^* complex stabilises cortical actin polymers

**Fig. 6.**
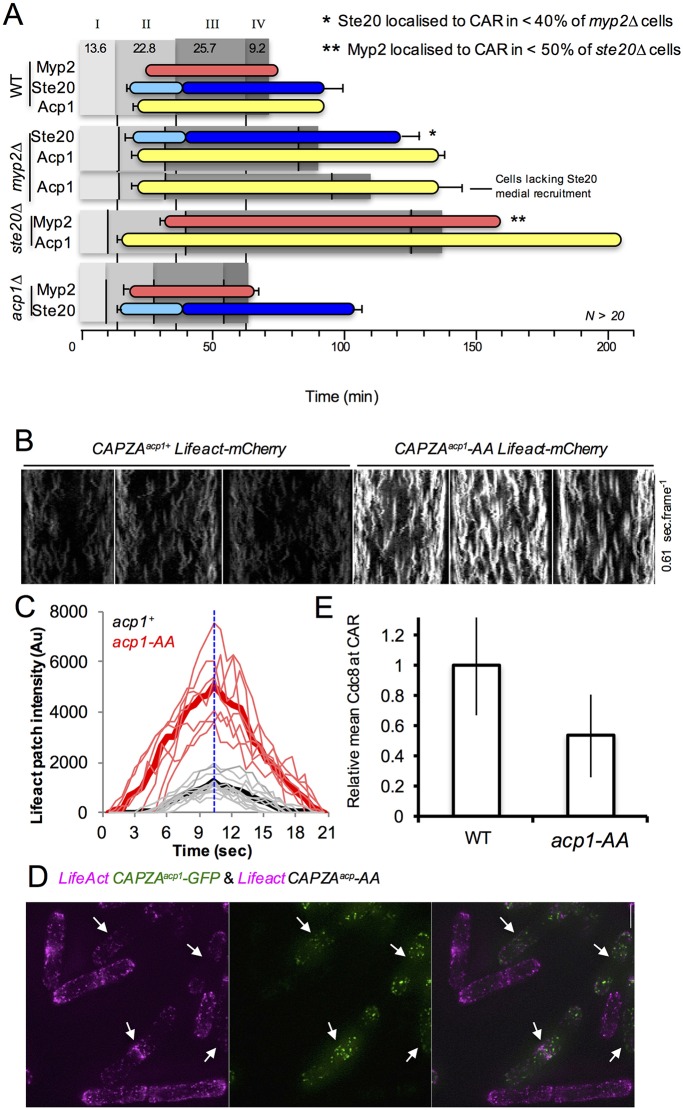
***CAPZA^a^^cp1^-AA* mutants disrupt actin dynamics and cytokinesis.** (A) The timing of the appearance of Myp2 ring (red bars), RICTOR^Ste20^ foci (light blue bars), Acp1 (yellow bars) medial recruitment and RICTOR^Ste20^ ring recruitment (dark blue) were determined in wild-type, *myp2*Δ, *Rictor^ste20^*Δ (*ste20*Δ) and *acp1*Δ strains in relation to the events determined in Fig. 2E. Recruitment of these proteins to the CAR were observed in less than 40% (*) or 50% (**) of these deletion strains. CAR dynamics and composition were followed in >20 cells for each strain. Early exponential prototrophs were used in each experiment. (B) mCherry kymographs generated from 100 timeframe maximum projections from 13 *z*-plane images of *CAPZA^acp1+^ Lifeact-mCherry* (left panels) and *CAPZA^acp1^-AA Lifeact-mCherry* (right panels) cells (0.6 s/frame). (C) Graph showing lifetime kinetics of Lifeact signal from individual (faint lines) actin patches and overall averages (thick lines) of *CAPZA^acp1+^ Lifeact-mCherry* (black lines) and *CAPZA^acp1^-AA* *Lifeact–mCherry* (red lines) cells. (D) Maximum projections of a mixture of *CAPZA^acp1^–GFP Lifeact–mCherry* and *CAPZA^acp1^–AA* *Lifeact–mCherry cells*. Overlaying the GFP (green) and mCherry (magenta) signals demonstrate the increase in actin signal at cortical actin patches in the *CAPZA^acp1^-AA* mutant compared to GFP-labelled wild type (arrows) cells. (E) Histograms illustrating mean±s.d. relative Cdc8 at the CAR in wild-type (WT) and *CAPZA^acp1^-AA* cells (*n*>30 per strain).

The fission yeast CAPZA^Acp1^–CAPZB^Acp2^ complex regulates actin dynamics and cytokinesis (Kovar et al., 2005; Nakano and Mabuchi, 2006), and is recruited to the cell equator during cell division (Movie 7). CAPZA^Acp1^–GFP remained localised at the cell equator at the end of cytokinesis for significantly longer in *Rictor^ste20^*Δ and *myp2*Δ cells than in wild-type cells (Fig. 6A). In contrast, the timing of RICTOR^Ste20^−3GFP CAR recruitment was unaffected in cells lacking the *CAPZA^acp1^* gene (Fig. 6A).

We next used LifeAct (Huang et al., 2012; Riedl et al., 2008) to compare actin dynamics in *CAPZA^acp1+^* and *CAPZA^acp1^*-*AA* mutant cells. Interestingly, the *CAPZA^acp1^-AA* allele, which stabilises the CAPZA^Acp1^–CAPZB^Acp2^ complex (Fig. 5D), had a substantial impact upon the stability of cortical filamentous actin patches (Fig. 6B–D). Actin patches were depolarised, had a longer lifetime, and increased polymerisation rates in *CAPZA^acp1^-AA* cells when compared to wild type (growth rates: wild-type, 220.1 arbitrary units (AU) s^−1^; *CAPZA^acp1^-AA*, 573.8 AU s^−1^; shrinkage rates: wild type, −167.5 AU s^−1^; *CAPZA^acp1^-AA*: −543.9 AU s^−1^) (Fig. S3C). Cells possessing the *CAPZA^acp1^-AA* allele had five times more LifeAct signal associated with cortical actin patches compared to wild type (Fig. 6B–D; Fig. S3C). In contrast, there was no significant difference in the number of cortical actin patches, patch-associated CAPZA^Acp1^ protein localisation or total CAPZA^Acp1^ protein within wild-type and mutant cells (Fig. S3C). This indicates the *CAPZA^acp1^-AA* allele does not affect actin nucleation or stability of the CAPZA^Acp1^ protein, but that it remains associated with actin polymers longer than wild type, which continue to accumulate actin at the uncapped end.

### *CAPZA^acp1^-AA* has aberrant CAR morphology

**Fig. 7.**
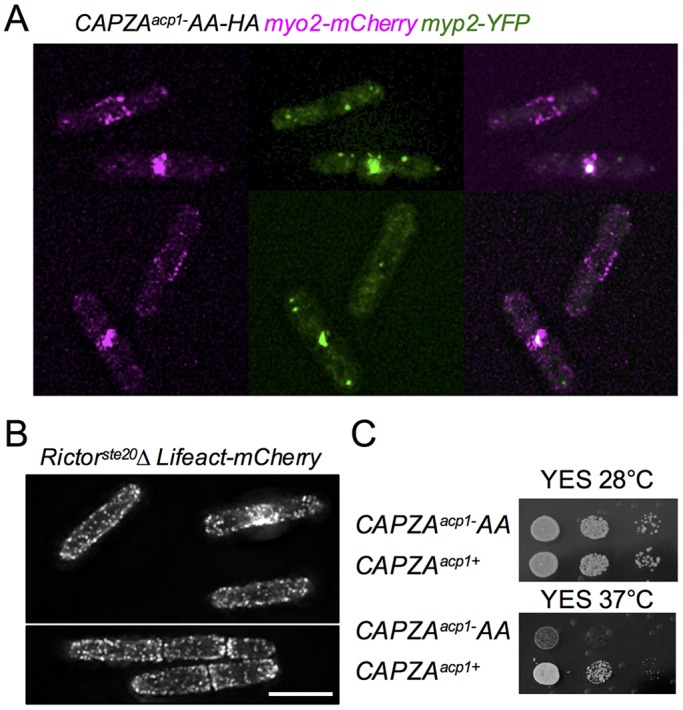
**TORC2 and CAPZA^a^^cp1^-AA mutants disrupt CAR and actin localisation.** (A) Micrographs of Myo2 (magenta) and YFP (green) signal *CAPZA^a^^cp1^-AA***–***HA myo2–mCherry YFP–myp2* cells. (B) Micrograph of mCherry signal from *Rictor^ste20^*Δ *Lifeact–mCherry* cells illustrating the cytokinesis defect and lack of polarised actin signal. Scale bar: 10 μm. (C) The CAPZA^Acp1^ phosphorylation site mutant *CAPZA^acp1−^AA* is sensitive to heat stress at 37°C.

It has been suggested that cells possess a finite pool of actin monomers, for which different actin nucleators (Arp2/3 and formins) compete (Burke et al., 2014; Suarez et al., 2015). Thus, increasing the proportion of the actin pool incorporated within Arp2/3-nucleated cortical actin patches would result in a reduction in the actin available for incorporation into formin-nucleated actin cables, which are essential for cytokinesis to occur. The amount of CAR-associated Cdc8 (the yeast tropomyosin) provides a specific measure of the amount of formin-nucleated actin cables (Skoumpla et al., 2007; Skau and Kovar, 2010). Consistent with the ‘finite pool of actin monomers’ model, quantification of the CAR-associated Cdc8 revealed a reduction in the amount of actin incorporated into Cdc12-nucleated (Cdc12 is a yeast formin) actin cables within the CAR in CAPZA*^acp1^-AA* cells (Fig. 6E). Therefore, although cortical actin patches are more stable and persist for longer in the *CAPZA^acp1^-AA* cells, there are fewer Cdc8-associated actin cables in the CAR. This might provide an explanation for the CAR instability in *CAPZA^acp1^-AA* cells (Fig. 7A). Thus, the aberrant CAR morphology phenotype observed in *CAPZA^acp1^-AA* mutant cells emulates that seen in *TORC2* deletion strains. Finally, in contrast to wild-type cells (Huang et al., 2012), and similar to with *CAPZA^acp1^-AA*, the actin cytoskeleton was depolarised in *Rictor^ste20^*Δ cells (Fig. 7B). This finding is consistent with the observed increased cell diameter of cells lacking TORC2 function (Fig. 1B), as cell growth might no longer be exclusively restricted to cell ends.

Finally, stress of wild-type cells at 37°C transiently disrupts actin organisation and dynamics, before a normal distribution is re-established and growth resumes within 90 min (Petersen and Hagan, 2005). TORC2 has a crucial role in responding to induced heat stress as TORC2-deficient cells are hypersensitive to transient increases in heat (Kawai et al., 2001; Weisman and Choder, 2001). Intriguingly, *CAPZA^acp1^-AA* cells also failed to recover from the stress imposed by a shift from 28°C to 37°C (Fig. 7C). CAPZA^Acp1^ protein levels did not change following heat stress of either wild-type or *CAPZA^acp1^-AA* cells (Fig. S3A). Hence, the mutations did not affect CAPZA^Acp1^ stability, and the alteration in actin dynamics might cause the observed increased sensitivity to heat stress in *CAPZA^acp1^-AA* cells.

In summary, we conclude that the TORC2-dependent phosphorylation of CAPZA^Acp1^ reduces the stability of the CAPZA^Acp1^–CAPZB^Acp2^ heterodimer. This in turn modulates the stability of cortical actin patches and alters the concentration of free monomeric-actin within the cell, to affect the timing and fidelity of cytokinesis.

## DISCUSSION

Here, we describe a novel TORC2 recruitment to the CAR that plays a role in maintaining the fidelity of cytokinesis in fission yeast. We describe a mechanism by which a myosin V and myosin II co-purify with TORC2 and play a role in its localisation to the CAR during cytokinesis. Each myosin appears to play a discrete role in affecting TORC2 CAR localisation. Myo51 ensures TORC2 is recruited to the CAR in the early stages of anaphase, whereas Myp2 maintains TORC2 at the CAR. Although TORC2 is required to maintain the CAR at the cell equator and prevent it from drifting along the cell cortex, both TORC2 and Myp2 maintain the integrity of a single CAR structure and prevent it from splitting in two during its constriction. It is currently unclear why RICTOR^Ste20^ only associates with the CAR in a subset of *myp2*Δ cells, however this might explain why cytokinesis defects are only observed in a subset of cells lacking this myosin II.

In the absence of TORC2 signalling, the stability and activity of the actomyosin ring and therefore cytokinesis are altered. These changes in actin dynamics arise in part from TORC2-dependent phosphorylation of the actin-capping protein CAPZA^Acp1^. Perturbation of CAPZA^Acp1^ phosphorylation increases the stability of the CAPZA^Acp1^–CAPZB^Acp2^ heterodimer (Fig. 5), significantly alters the cellular organisation of the actin cytoskeleton (Fig. 6) and disrupts CAR function (Fig. 7A). The increased severity in growth and cytokinesis phenotypes of *Rictor^ste20^*Δ cells compared to the CAPZA*^acp1^-AA* and *myp2*Δ mutants (Figs 1, 3 and 7) suggest that CAPZA^Acp1^ is very unlikely to be the sole protein with a key role in cytokinesis that is regulated in a TORC2-dependent manner. Consistent with this view, an increase in myosin II Myo2 levels was seen in the *Rictor^ste20^*Δ cells but not in *acp1*Δ or *myp2*Δ cells (data not shown), thus TORC2 regulates Myo2 in an as yet unidentified manner. Multiple potential TORC2-regulated proteins involved in coordinating cell division co-purified with TORC2 (Fig. 2A). However, our SILAC analysis to date has not identified phospho-peptides that showed differential phosphorylation upon Torin1 inhibition of TOR function in any of the co-purifying proteins. Crucially, we recently demonstrated that the co-purifying SCYL family pseudo-kinase Ppk32 is a novel regulator of TOR signalling, and intriguingly Ppk32 concentrates at the cell equator during cell division in more than 60% of cells (Kowalczyk and Petersen, 2016). Thus, the TORC2-dependent regulation of CAPZA^Acp1^–CAPZB^Acp2^ that we describe here is likely to be one of multiple TORC2-dependent mechanisms regulating the fidelity and timing of cytokinesis and cell division.

The TORC2 control of CAPZA^Acp1^–CAPZB^Acp2^ heterodimer stabilisation enables cells to couple actin stability with changes in cell growth and division, which can be implemented in response to environmental stress. Crucially, this TORC2-dependent change in CAPZA^Acp1^–CAPZB^Acp2^ affinity and actin stability provides an explanation for the enhanced F-actin cable stability observed in TORC2-deficient cells (Ikai et al., 2011; Matsuo et al., 2007), which appears to be key in ensuring cell survival following environmental stress. Similar controls are likely to underlie TORC2 control of actin dynamics and cytokinesis in mammalian cells, as knockout of TORC2 components in HeLa cells leads to similar increases in the abundance of actin fibres and increased cytoplasmic paxilin association (Sarbassov et al., 2004). Furthermore, serine 2481 auto-phosphorylated mTOR localises to the cleavage furrow at the onset of cytokinesis (Vazquez-Martin et al., 2009), suggesting that active mTOR plays a conserved role in cytokinesis and cell division.

## MATERIALS AND METHODS

### Strains and cell cultures

Strains used in this study are listed in Table S1. Unless otherwise specified, cells were cultured at 28°C in Edinburgh minimal media (EMM2) (Fantes, 1977) using 20 mM L-Glutamic acid as a nitrogen source (EMMG). Cells were grown exponentially for 48 h before being harvested or examined microscopically at early exponential phase of 1.5×10^6^ cells/ml. *Rictor^ste20^*Δ cells were frozen immediately after spore germination. Cells were tested for sensitivity to stress and sterility to ensure no suppressors had accumulated. All *Rictor^ste20^*Δ cell cultures were maintained in exponential growth and never exposed to starvation.

### FACS analysis

*S. pombe* DNA content was measured by flow cytometry as previously described (Costello et al., 1986).

### Large scale Tor1 immunoprecipitation

Wild-type cells (JP350) were grown in EMMG to 2.5×10^6^ cells/ml (5 l per immunoprecipitation condition) and harvested and disrupted using a freezer mill (SPEX 6870) in liquid nitrogen. The cell powder was thawed in immunoprecipitation buffer (50 mM HEPES pH 7.5, 150 mM NaCl, 0.1% CHAPS, 50 mM L-arginine, 50 mM L-glutamic acid, 0.05% Tween 20, 120 mM β-glycerophosphate di-sodium salt, 4 mM Na_3_VO_4_, 100 mM NaF, 2 mM PMSF, 10 mM N-ethylmaleimide, 2 mM dithiothreitol and 2×Roche EDTA-free protease inhibitor cocktail). The cleared supernatant was incubated with Invitrogen protein G Dynabeads, pre-incubated with anti-Tor1 antibodies (5 µg; Hálová et al., 2013) or no antibody for control, for 60 min at 4°C. Beads were then washed twice with immunoprecipitation buffer plus 50 mM NaCl (200 mM final concentration) and proteins were eluted by heating at 80°C for 10 min. The samples were loaded on to a NUPAGE Bis-Tris 4–12% gel (Life Technologies). The gel was fixed with 7% acetic acid and 25% methanol and stained with Coomassie Brilliant Blue. The entire lane was cut into small bands, before being sent for protein identification. Mass spectrometry data were analysed by Scaffold™ 3 software.

### Immunoprecipitation of the Myo51 cargo-binding domain

Immunoprecipitation and cell lysis was performed in the following buffer – 50 mM HEPES pH7.5, 150 mM NaCl, 0.1% CHAPS, 50 mM L-arginine, 50 mM L-glutamic acid, 0.05% Tween, 50 mM NaF, 2 mM Na_3_VO_4_, 60 mM β-glycerophosphate di-sodium salt, 5 mM N-ethylmaleimide, 1 mM PMSF, 10 μM Z-LLF 1 mM DTT and 1× protease inhibitor without EDTA (Complete, Mini; Roche), with the addition of 150 mM NaCl (300 mM final concentration) and 100 mM KCl for washes. 6×10^8^ cells, harvested at a density of 2×10^6^/ml, were used per immunoprecipitation. Protein A dynal beads (Life Technologies) were used and the cell lysis solution was pre-cleared for 10 min with a bead-only slurry. Cleared extracts were incubated with GFP monoclonal antibody (clones 7.1 and 13.1, Roche) pre-coated dynal A beads for 30 min at 4°C and washed five times prior to elution from beads in loading buffer at 70°C for 15 min.

### SILAC cell culture

*car2::NAT lys1-131 arg3-d4* cells were inoculated in YES medium overnight and then washed into EMM-G containing 75 mg/l of either light [L-arginine monohydrochloride (Sigma) and L-lysine monohydrochloride (Sigma)] or medium [lysine-L, 2HCl 4.4.5.5-D4 (Cat code DLM-2640, Eurisotop), arginine-L, HCl, U-13C6 99%13C (cat. no. CLM-2265, Eurisotop)] amino acids. Cells were cultured in log phase for 48 h to ensure complete incorporation of labelled amino acids into the proteome. Light-labelled cultures were treated with DMSO and medium-labelled cultures were treated with a final concentration of 25 µM Torin1 at a density of 2.04×10^6^ cells/ml. Approximately 4.8×10^9^ cells were harvested for each sample. After 30 min cultures were harvested by centrifugation (3000 ***g*** for 5 min), washed in 20 ml of STOP buffer (10 mM EDTA, 1 mM sodium azide, 50 mM sodium fluoride, 0.9% NaCl), followed by washing with 10 ml of ice cold ddH_2_O. The final pellets were then resuspended in an appropriate volume of ice cold ddH_2_O and dropped directly into liquid nitrogen to produce frozen cell droplets.

### SILAC protein extraction

Samples were processed using a SPEX Sample Prep LLC 6850 Freezer Mill in presence of liquid nitrogen. The resulting cell powder was resuspended in denaturation buffer (6 M urea, 2 M thiourea, 1% n-octyl glucoside) at a ratio of 500 mg powder to 500 µl denaturation buffer. Insoluble material was removed by centrifugation (13,000 ***g***, 10 min at 4°C) and the supernatant was designated supernatant I (soluble fraction). The pellet was then resuspended in 500 µl denaturation buffer, 500 µl glass beads were added and then subjected to 20 s shaking in a FastPrep machine (FP120, Qbiogene). The resulting suspension was again centrifuged (13,000 ***g***, 10 min at 4°C) and the supernatant retained (supernatant II). The pellet was then discarded. Protein concentrations were determined by Bradford assay according to the manufacturer's instructions.

### Mass spectrometry for SILAC

Respective supernatants I and II derived from the ‘light’ and ‘medium’ labelled cell cultures were combined and proteins were precipitated at −20°C using ice-cold acetone in methanol left on ice overnight. The proteins were pelleted by centrifugation (2200 ***g***, 20 min, 4°C) and washed with 80% ice-cold acetone. Dried proteins were resolved in digestion buffer (6 M urea, 2 M thiourea, 10 mM Tris-HCl, pH 8.0) and mixed at a 1:1 ratio according to measured protein amounts. The mixtures were digested in solution with trypsin as described previously (Borchert et al., 2010). For proteome analyses, 100 µg of the mixtures were fractionated by isoelectric focusing on an OffGel 3100 Fractionator (Agilent) according to the manufacturer's instructions. Focusing was performed using 13-cm (12 well) Immobiline DryStrips pH 3–10 (Bio-Rad) at a maximum current of 50 µA for 24 kVh. Peptide fractions were collected and desalted separately using C18 StageTips (Rappsilber et al., 2007).

For phosphoproteome analyses, 8 mg of each peptide mixture was subjected to phosphopeptide enrichment as described previously (Olsen et al., 2005) with minor modifications: peptides were separated by strong cation-exchange (SCX) chromatography with a gradient of 0 to 35% SCX solvent B resulting in seven fractions that were subjected to phosphopeptide enrichment by TiO_2_ beads. Elution from the beads was performed three times with 100 µl of 40% ammonia hydroxide solution in 60% acetonitrile (pH >10.5). Fractions rich in peptides were subjected to multiple TiO2 enrichment. Enrichment of phosphopeptides from the SCX flow-through was completed in five cycles.

Liquid chromatography tandem mass spectrometry (LC-MS/MS) analyses were performed on an EasyLC nano-HPLC (Proxeon Biosystems) coupled to an LTQ Orbitrap XL (Thermo Scientific) for phosphopeptide analyses, or an LTQ Orbitrap Elite mass spectrometer (Thermo Scientific) for proteome analyses as described previously (Koch et al., 2011). The peptide mixtures were injected onto the column in HPLC solvent A (0.5% acetic acid) at a flow rate of 500 nl/min and subsequently eluted with a 87-min (proteome) or a 127-min (phosphoproteome) segmented gradient of 5%, 33% and 90% HPLC solvent B (80% acrylonitrile in 0.5% acetic acid). During peptide elution, the flow rate was kept constant at 200 nl/min. For proteome analysis, the 20 most intense precursor ions were sequentially fragmented in each scan cycle. For the phosphoproteome analysis, the five most intense precursor ions were fragmented by multistage activation of neutral loss ions at −98, −49, and −32.6 Th relative to the precursor ion (Schroeder et al., 2004). In all measurements, sequenced precursor masses were excluded from further selection for 90 s. Full scans were acquired at resolution of 60,000 (Orbitrap XL), or 120,000 (Orbitrap Elite). The target values were set to 5000 charges for the LTQ (MS/MS) and 10^6^ charges for the Orbitrap (MS), respectively; maximum allowed fill times were 150 ms (LTQ) and 1000 ms (Orbitrap). The lock mass option was used for real-time recalibration of mass spectrometry spectra (Olsen et al., 2005).

The mass spectrometry data of all SILAC experiments were processed using default parameters of the MaxQuant software (v1.2.2.9) (Cox and Mann, 2008). Extracted peak lists were submitted to database search using the Andromeda search engine (Cox et al., 2011) to query a target-decoy (Elias and Gygi, 2007) database of *S. pombe* proteome (http://www.pombase.org/, Protein Dataset in FASTA format, downloaded on the 6 April 2011), containing 5076 protein entries and 248 commonly observed contaminants.

### Molecular manipulations and generation of single point mutations

The *CAPZA^acp1^*Δ base strain was constructed from a DNA cassette for *acp1* deletion was prepared by PCR amplification. This construct was used to replace the *acp1^+^* gene at the native locus with the *nat^+^*. To generate the *acp1-S172A-S189A* point mutation, standard site-directed mutagenesis was used. The mutant acp1 was then used to replace the *nat1 rpl42^+^* gene in JP2222 (Fennessy et al., 2014). The resulting strains were back-crossed and prototroph progeny was selected. The presence of the mutant *acp1* allele was verified by PCR. Thus, all *acp1* single point mutations used in this study are integrated into the *acp1* locus, and are all prototroph strains. C-terminal tagging method was performed as previously described (Bähler et al., 1998) using *acp1* C-terminal-specific primers.

### Acp1–HA immunoprecipitations

Immunoprecipitation of Acp1–HA was carried out under non-denaturing conditions allowing for pulldown of any bound Acp2–GFP. Cell lysis buffer consisted of 50 mM HEPES pH 7.5, 100 mM KCl, 50 mM NaCl, 0.2% Tween 20, 0.1 mM EDTA, 1× protease inhibitors (Complete, Mini; Roche), 25 mM NaF, 2 mM Na_3_VO_4_, 25 mM β-glycerophosphate di-sodium salt, 5 mM N-ethylmaleimide, 0.5 mM PMSF, with the addition of a further 25 mM NaCl and 0.25% CHAPS in wash buffer. Briefly, anti-HA antibody (F7 clone, Santa Cruz Biotechnology) was dimethyl pimelimidate cross-linked to Dynal beads protein A (Life Technologies) and incubated with lysed *S. pombe* cells (3×10^8^ cells per immunoprecipitation, harvested at 1.5×10^6^cells/ml) for 1 hour at 4°C, followed by five washes with wash buffer. Bound proteins were eluted in loading buffer (100 mM Tris-HCl pH 6.8, 4% SDS, 0.2% Bromophenol Blue, 20% glycerol and 100 mM DTT) at 70°C for 15 min then loaded onto 12% tris-glycine PAGE gels, transferred to PVDF membrane and the membrane cut at ∼45 kDa enabling detection of both CAPZA^Acp1^–HA and CAPZB^Acp2^–GFP on the same gel.

### Western blotting

The trichloroacetic acid (TCA) precipitation protocol was followed for total protein extracts (Caspari et al., 2000). The following dilutions of antibodies were used in this study: 1:1000 anti-Myo2 (Coulton et al., 2010) 1:100 anti-GFP (Roche, #11814460001), 1:2000 anti-HA (Santa Cruz Biotechnology, sc-7392 F7 clone). Alkaline-phosphatase-coupled secondary antibodies were used for all blots followed by direct detection with NBT/BCIP (VWR) substrates on PVDF membranes.

### Microscopy

Samples were visualised using an Olympus IX71 microscope with a PlanApo 100× OTIRFM-SP 1.45 NA lens mounted on a PIFOC *z*-axis focus drive (Physik Instrumente, Karlsruhe, Germany), and were illuminated using LED light sources (Cairn Research Ltd, Faversham, UK) with appropriate filters (Chroma, Bellows Falls, VT). An Optosplit device (Cairn Research Ltd) was used to allow simultaneous acquisition of signals from two fluorophores that emitted light of different wavelengths. Samples were visualised using either a QuantEM (Photometrics) or ProEM 1024B (Princeton Instruments) EMCCD camera, and the system was controlled with Metamorph software (Molecular Devices). Each 3D-maximum projection of volume data was calculated from 21 *z*-plane images, each 0.2 µm apart, and analysed using Metamorph and Autoquant X software. During live-cell imaging, cells were cultured in EMMG. Cells were grown exponentially at 25°C for 48 h before being mounted (without centrifugation) onto lectin-coated (Sigma L2380; 1 mg/ml) coverslips with an a Bioptechs FCS2 (Bioptechs, Butler, PA), fitted onto an ASI motorised stage (ASI, Eugene, OR) on the above system, with the sample holder, objective lens and environmental chamber held at the required temperature. Cdc8 immunofluorescence was undertaken using conditions described previously (Skoumpla et al., 2007). Mean cell width, septa positioning and nuclear diameter was determined from measurements of ∼300 cells for each strain. In the determining of the timing of CAR formation and constriction, and protein recruitment in wild-type and mutant strains, time-lapse imaging and subsequently analysis was undertaken on >20 cells for each individual strain.
